# Electroconvulsive therapy for Patients with Intellectual Disability. When and how?

**DOI:** 10.1192/j.eurpsy.2022.1913

**Published:** 2022-09-01

**Authors:** F. Azevedo, R. André, I. Donas-Boto, D. Jeremias, C. Almeida

**Affiliations:** 1 Centro Hospitalar de Lisboa Ocidental, Psychiatry, Lisbon, Portugal; 2 Centro Hospitalar Universitário Lisboa Norte, Psychiatry, Lisboa, Portugal; 3 Centro Hospitalar de Lisboa Ocidental, Psychiatry Department, Lisboa, Portugal; 4 NOVA Medical School, Psychiatry And Mental Health, Lisboa, Portugal

**Keywords:** Electroconvulsive therapy, Intellectual Disabilty, Neurodevelopment, ECT

## Abstract

**Introduction:**

Intelectual disability is an illness with an important burden on patients and caregivers, especially when severe and when comorbidities such as other psychiatric disorders are present. There are case reports of treatment resistant self-aggression, agitation, epilepsy, catatonia and psychosis successfully treated with electroconvulsive therapy although controlled studies were not found.

**Objectives:**

This work reviewed the current evidence for the use of electroconvulsive therapy in the management of patients with intellectual disability as well as its ethical and methodological implications.

**Methods:**

Non-systematic review of the literature with selection of scientific articles published in the past 20 years; by searching Pubmed and Medscape databases using the combination of MeSH descriptors. The following MeSH terms were used: “electroconvulsive therapy”, “intellectual disability”.

**Results:**

Patients with intellectual disability can have incapacitating comorbilities that greatly impair quality of life, and may require withdrawl from the community Treatment often differs from the general population as psychotropic medication can worsen other comorbilities. Electroconvulsive therapy can be a relevant treatment option for comorbidities in this population due to its safety profile. Ethical considerations should be taken into account, especially with non-verbal patients or when adequate representatives have not been chosen or cannot be reached. Different legal challenges may be present on different countries.

**Conclusions:**

Electroconvulsive therapy and intellectual disability share the burdens of heavy stigma and low investment. Intellectual disability and it’s commorbidites present both a diagnostic and treatment challenge. Electroconvulsive therapy is an important weapon capable of restoring patients to their families and diminishing the burdens of caregivers and healthcare systems

**Disclosure:**

No significant relationships.

